# A Mutation in Mouse *Pak1ip1* Causes Orofacial Clefting while Human *PAK1IP1* Maps to 6p24 Translocation Breaking Points Associated with Orofacial Clefting

**DOI:** 10.1371/journal.pone.0069333

**Published:** 2013-07-25

**Authors:** Adam P. Ross, M. Adela Mansilla, Youngshik Choe, Simon Helminski, Richard Sturm, Roy L. Maute, Scott R. May, Kamil K. Hozyasz, Piotr Wójcicki, Adrianna Mostowska, Beth Davidson, Iannis E. Adamopoulos, Samuel J. Pleasure, Jeffrey C. Murray, Konstantinos S. Zarbalis

**Affiliations:** 1 Department of Pathology and Laboratory Medicine, University of California Davis, Sacramento, California, United States of America; 2 Department of Pediatrics, University of Iowa, Iowa City, Iowa, United States of America; 3 Department of Neurology, University of California San Francisco, San Francisco, California, United States of America; 4 Interdisciplinary Center for Neurosciences, Heidelberg University, Heidelberg, Germany; 5 Institute for Cancer Genetics, Columbia University, New York City, New York, United States of America; 6 Molecular Neurobiology Laboratory, The Salk Institute for Biological Studies, La Jolla, California, United States of America; 7 Department of Pediatrics, Institute of Mother and Child, Warsaw, Poland; 8 Department of Plastic Surgery, Wrocław Medical University, Polanica Zdroj, Poland; 9 Department of Plastic Surgery, Medical Centre, Polanica-Zdrój, Poland; 10 Department of Biochemistry and Molecular Biology, Poznan University of Medical Sciences, Poznan, Poland; 11 Department of Internal Medicine, University of California Davis, Sacramento, California, United States of America; 12 Institute for Pediatric Regenerative Medicine, Shriners Hospitals for Children Northern California, Sacramento, California, United States of America; University of Houston, United States of America

## Abstract

Orofacial clefts are among the most common birth defects and result in an improper formation of the mouth or the roof of the mouth. Monosomy of the distal aspect of human chromosome 6p has been recognized as causative in congenital malformations affecting the brain and cranial skeleton including orofacial clefts. Among the genes located in this region is *PAK1IP1*, which encodes a nucleolar factor involved in ribosomal stress response. Here, we report the identification of a novel mouse line that carries a point mutation in the *Pak1ip1* gene. Homozygous mutants show severe developmental defects of the brain and craniofacial skeleton, including a median orofacial cleft. We recovered this line of mice in a forward genetic screen and named the allele *manta-ray* (*mray*). Our findings prompted us to examine human cases of orofacial clefting for mutations in the *PAK1IP1* gene or association with the locus. No deleterious variants in the *PAK1IP1* gene coding region were recognized, however, we identified a borderline association effect for SNP rs494723 suggesting a possible role for the *PAK1IP1* gene in human orofacial clefting.

## Introduction

Among the most common birth defects are clefts of the lip and/or palate (CL/P), which affect about one in 700 births worldwide [1], [2]. Orofacial defects can be syndromic, with a variety of other clinical features, or isolated (approximately 70%) and can involve major difficulties with food intake, speech, hearing and ensuing psychological development. As a subset of orofacial clefts, median clefts are extremely rare and originate from the failure of the frontonasal processes to fuse along the midline during development. Orofacial clefts have a complex and poorly understood etiology with both genetic and environmental factors contributing. Some progress in the identification of genes implicated in CL/P disorders has occurred over the last decade, with a major contribution for new candidate genes in recent years from Genome Wide Association Studies (GWAS) [3]–[5]. Nevertheless, variants in these genes (e.g. *IRF6*, *MAFB, ARGHAP29, VAX1,* 8q24) do not account for all cases with genetic etiology underlining the need for identifying new genes contributing to the burden of orofacial clefts and the molecular processes they regulate [2], [6].

One chromosomal region of importance for orofacial clefts is 6p24 (orofacial cleft 1, OMIM 119530) with several studies suggesting an associated locus. Chromosomal translocation breaking points in patients with orofacial clefts have been finely mapped to 6p24.3 [7], [8]. *TFAP2A* lies within this region; mutations in this gene, which encodes a transcription factor, cause branchio-ocular-craniofacial syndrome, an autosomal dominant disorder that includes CL/P [9]. In addition, TFAP2A regulates through a specific binding site the expression of *IRF6,* causative in Van der Woude syndrome, which can include CL/P. Interestingly, this binding site contains a variant (rs642961) that disrupts TFAP2A binding and is overtransmitted in clefting patients [10]. Additional genes, distal to the translocation breaking points, include several important regulators of developmental processes such as *BMP6* and several members of the *FOX* gene family, but none has been with certainty associated with orofacial clefts. In addition, several studies have shown linkage of CL/P syndromes to markers in 6p23 [11]–[14].

*PAK1IP1* (Pak1 interacting protein 1), maps to 6p24.2 approximately 203 kb centromeric from OFC1. The encoded protein is also known as hPIP1 (human Pak1 Interacting Protein 1) or as the yeast homolog Skb15 (*Schizosaccharomyces pombe* Shk1 kinase binding protein 15) and was initially identified in two independent yeast two-hybrid screens using yeast and human Pak1 (P21-activated kinase) as bait respectively [15], [16]. P21-activated kinases (PAKs) are serine-threonine protein kinases that are positively regulated by the small GTPases, Cdc42 and Rac1 [17], [18]. In vertebrates PAKs control diverse cellular processes, including gene expression, cytoskeletal actin and tubulin assembly, neurite outgrowth, cell cycle control, and apoptosis. Pak1ip1 is largely comprised of WD40 repeats and was initially described as a negative regulator of Pak1. Interestingly, more recent research has shown that Pak1ip1 also localizes to the nucleolus where it is involved in ribosome biogenesis, in light of which its interaction with Pak1 and the cellular events it mediates through this function appear secondary [19]. By facilitating ribosomal RNA processing, Pak1ip1 plays a regulatory role in cell growth and proliferation apparently through direct interaction with the tumor protein 53 (Tp53)-murine double minute 2 (Mdm2) loop upon cellular stress [20]. Furthermore, by causing craniofacial anomalies if mutated, Pak1ip1 joins a group of molecules causative in ribosomopathies associated with craniofacial defects. They include for instance TCOF1 (Treacher-Collins-Franceschetti syndrome 1) and ribosomal proteins S19 and S24 (RPS19, RPS24) in Diamond-Blackfan anemia [21], [22].

Here, we demonstrate that Pak1ip1 is a factor required for proper craniofacial morphogenesis presumably by its unique role in ribosome biogenesis, further adding to our understanding of craniofacial morphology and its underlying molecular regulation.

## Results

### *manta-ray*, a novel mouse line with orofacial clefting

**Figure 1:**
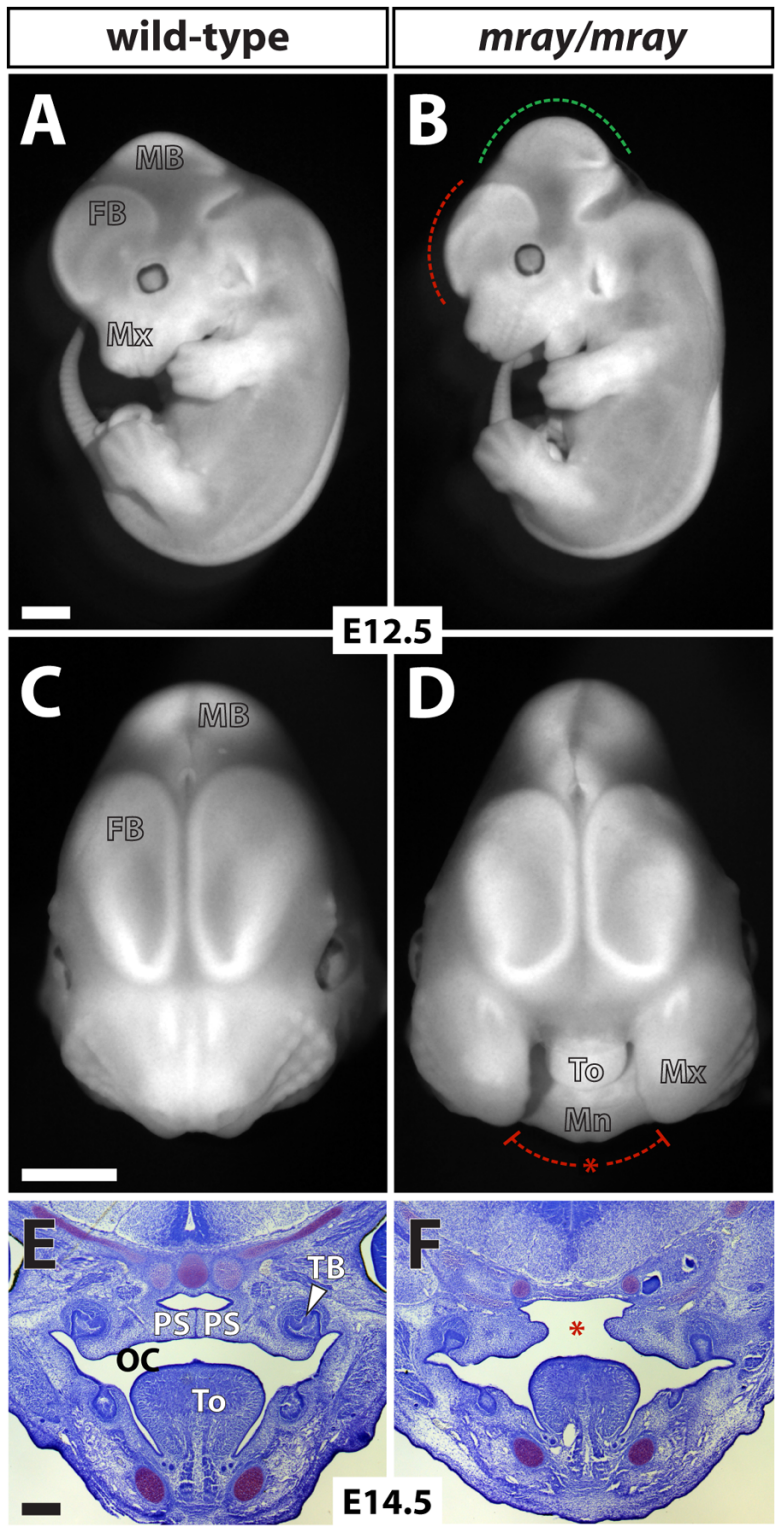
Homozygous *mray* mutants show severe craniofacial malformations starting early in development. **A, B,** Fluorescent images of a wild-type embryo compared to a homozygous *mray* littermate at E12.5. The dashed lines indicate the relative decrease in the size of the forebrain (red) but increase in the size of the midbrain in the mutant (green) respectively. In **C** and **D** frontal views of the head photographed under fluorescent lighting illustrate the median cleft of affected mutants. **E, F,** Nissl-stained coronal sections of the oral cavity of 14.5 day old embryos show that the palatal shelves in homozygous *mray* mutants fail to sufficiently grow out and eventually fuse. FB: forebrain, MB: midbrain, OC: oral cavity, PS: palatal shelf, TB: tooth bud, To: tongue. Scale bars in **A–D** are 1 mm and in **E** and **F** 250 μm.

**Figure 2:**
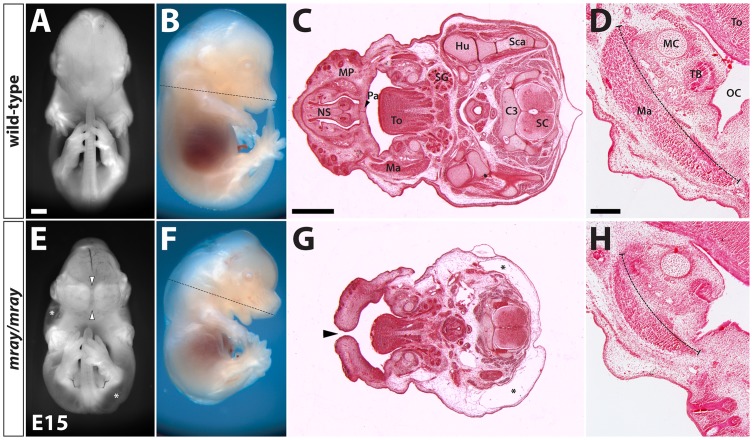
Midline clefting and reduced growth in *mray/mray* mutants. Frontal and side-views of an E15 wild-type embryo (**A, B**) compared to a homozygous *mray* littermate (**E, F**). The mutant is overall smaller and in the frontal view in **E** the median cleft (arrowheads) and large-scale edema (asterisks) covering almost the entirety of the body except for head and limbs can be appreciated. The dashed lines in the lateral views in **B** and **F** indicate the approximate positions of the sections in **C** and **G** respectively. **C, G** Hematoxylin-eosin-stained horizontal sections through the head and upper body show morphological changes in the homozygous *mray* mutant. The maxillary processes (MP) fail to fuse at the midline (arrowhead in **G**) resulting in a median cleft. Midline structures such as the nasal septum are absent and the back is covered by a skin edema (asterisks). Horizontal sections in D and H illustrate the severe hypoplasia of the masseter muscle in the mutant. C3 cervical vertebra 3, FB: forebrain, Hu: humerus, Ma: masseter muscle, MC: Meckel's cartilage, MB: midbrain, OC: oral cavity, Pa: secondary palate, SC: spinal cord, Sca: scapula, SG: salivary gland, TB: tooth bud, To: tongue. Scale bars are 1 mm in A and C and 200 μm in D.

In a forward genetic screen aimed at identifying mutations affecting neural development in mice [23], we recovered a mutant line with wide-ranging defects including median orofacial clefting. The mutation displayed autosomal-recessive inheritance and we named the line *manta-ray* (*mray*) due to the pathological morphology resulting from the clefting of the maxillae and secondary palate of the homozygous mutants (figure 1). At embryonic day (E)14.5 the palatal shelves of homozygous mutants elevate but fail to meet and fuse at the midline. Midline structures of the frontonasal skeleton, such as the nasal septum are severely reduced. Brain defects included a considerably smaller forebrain and hindbrain, which in part can be attributed to smaller ventricles (figure 1 and data not shown) but a relatively enlarged midbrain. In addition, most affected mutants are overall smaller at all stages, appear developmentally delayed, and a subcutaneous edema, possibly due to a heart and/or lymphatic defect, covers almost the entirety of the body (figure 2). The hypoplasia of craniofacial structures affects also the developing musculature as a comparison between the masseter muscles of wild-type and homozygous mutants demonstrates (figure 2). While *mray* homozygous mutants die as embryos, usually at around 14 days of gestation, heterozygotes appear phenotypically unaffected, are fertile, and have a normal lifespan.

**Figure 3:**
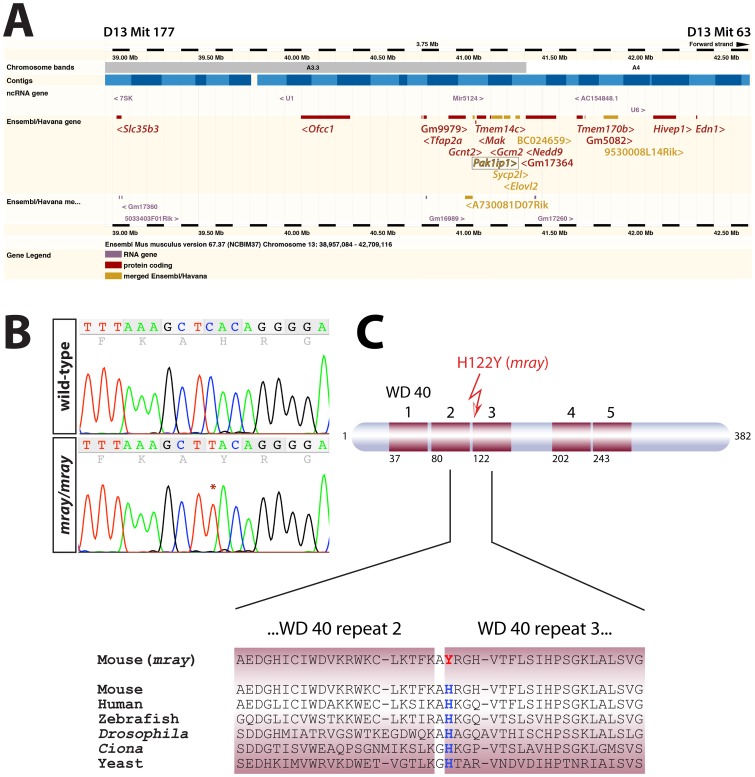
Positional mapping and identification of the causative mutation in the *Pak1ip1* gene. **A**, Ensembl-generated (version 67.37, NCBIm37) image of the genomic interval between markers D13Mit177 and D13Mit 63 the *manta-ray* (*mray*) mutation was mapped to. The coding sequences of all Ensembl/Havana genes shown in this diagram were sequenced but only one mutation was found, in the *Pak1ip1* gene (boxed). The sequenced genes list from proximal to distal as follows: *Slc35b3*, *Ofcc1*, Gm9979, *Tfap2a*, *Gcnt2*, A730081D07Rik, *Pak1ip1*, *Tmem14c*, *Mak*, *Gcm2*, *Sycp2l*, *Elovl2*, BC024659, GM17364, *Nedd9*, *Tmem170b*, Gm5082, 9530008L14Rik, *Hivep1*, *Edn1*. Angled brackets indicate gene directionality. **B**, Sequence chromatograms of wild-type and *mray* PCR amplicons showing the position of the mutation (C to T) in the trace. **C**, Schematic structure of the Pak1ip1 protein with WD 40 repeats indicated as red boxes and the first residue of every WD 40 repeat indicated below. The mutation leads to the substitution of a histidine residue by a tyrosine residue in the first position of the third WD 40 repeat. This position of the protein is entirely conserved as shown by the sequence alignment of six species below.

Employing microsatellite-based positional mapping and scoring more than 1500 meioses from separate crosses (heterozygote carriers, affected, and non-affected embryos), we confined the *mray* mutation to a 3.75 Mb-interval on chromosome 13 between markers D13Mit177 and D13Mit63 containing 20 protein-coding genes (list in figure 3). We sequenced all coding sequences in these genes and identified only one base pair substitution (C to T transition) in the *Pak1ip1* gene not present in the parental strains (C57BL/6J and FVB/NJ). The mutation leads to the substitution of a single amino acid residue from a histidine to a tyrosine in an entirely conserved position of the protein as sequence alignments of all known orthologs show (figure 3 and data not shown). Consequently, the *mray* allele presents the first mutant *Pak1ip1* allele described in mice, rendering this mouse line a very valuable model to study the effects of Pak1ip1 inactivation *in vivo*.

### Expression of *Pak1ip1* and midfacial specification in affected *manta-ray* mutants

**Figure 4:**
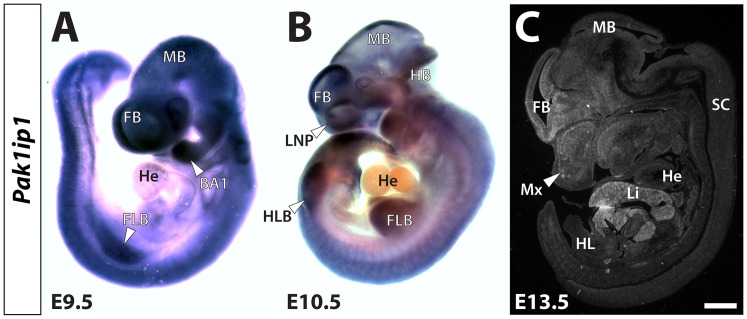
Developmental expression of the *Pak1ip1* gene. Expression analysis of *Pak1ip1* at midgestational time points ranging from E9.5 to E13.5 uncovers wide-ranging expression of the mRNA. Whole-mount RNA *in situ* hybridization on E9.5 (**A**) and E10.5 (**B**) embryos reveals that *Pak1ip1* is widely expressed throughout the embryo. Slightly higher expression levels can be seen in the brain, limbs, and facial primordia. Almost no transcripts can be detected in the heart of either stage. In **C** RNA *in situ* hybridization with a radiolabelled (^35^S) *Pak1ip1* probe on parasagittal section of an E13.5 embryo shows low-level expression in most tissues but higher transcript levels in forebrain, facial mesenchyme and particularly liver. BA 1: first branchial arch, FB: forebrain, FLB: forelimb bud, HB: hindbrain, He: heart, HLB: hindlimb bud, Li: liver, LNP: lateral nasal prominence, MB: midbrain, Mx: maxillary process, SC: spinal cord. Scale bar in **C** is 1 mm.

To better assess the role of Pak1ip1 during development and identify clues as to how its inactivation may result in developmental defects, we observe in affected *mray* embryos, we examined its expression during various embryonic stages. We conducted RNA *in situ* hybridization analysis on whole-mount embryos and sections of embryonic tissue. Our results show that *Pakip1* is widely expressed in the embryo, consistent with its role in ribosome biogenesis on which every cell depends. Specifically, *Pak1ip1* is expressed throughout the embryo with somewhat higher levels of expression in forebrain, facial primordia, limb buds, and liver (figure 4). Interestingly, the higher expression levels of Pak1ip1 in forebrain, facial structures, and liver, correspond to some of the most severely affected areas of the *mray/mray* embryos. These findings may point towards a particularly important role for Pak1ip1 expression levels in the histogenesis of certain organs.

**Figure 5:**
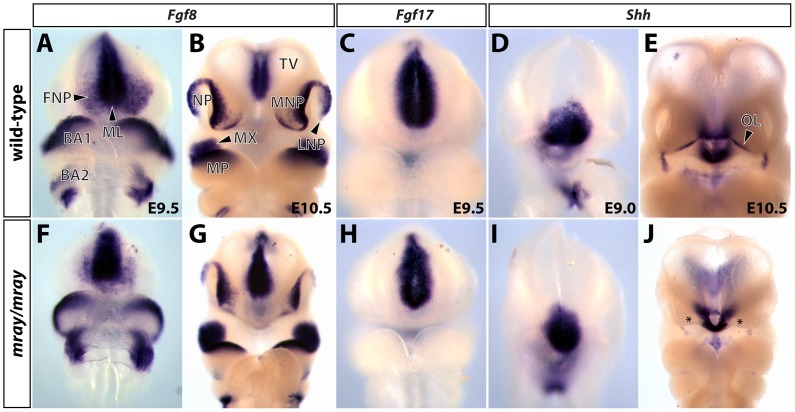
Midfacial specification in *mray/mray* embryos shows only mild changes compared to wild-type littermates. Whole-mount RNA *in situ* hybridizations reveal expression domains of *Fgf8* (**A, B F, G**), *Fgf17* (**C, H**), and *Shh* (**D, E, I, J**) in wild-type and mutant embryos at stages E9.0 to E10.5. Only mild changes in the midfacial expression domains of these genes can be seen in homozygous *mray* mutants, suggesting an overall proper specification of the midface. The slightly smaller expression domains of all analyzed genes in the E9.0 and E9.5 *mray/mray* embryos correlate well with overall smaller size of these embryos. Particularly, at E10.5 the overall smaller size and developmental delay of the mutant embryos becomes apparent (**G, J**). In the mutant, considerably lower *Shh* expression along the ectodermal odontogenic line is probably due to general developmental delay and marked by asterisks in **J**. BA1, 2: branchial arch1, 2, FNP: frontonasal prominence, LNP: lateral nasal process, ML: midline, MP: mandibular process, MNP: medial nasal prominence, MX: maxillary process, NP: nasal pit, OL: odontogenic line, TV: telencephalic vesicle.

Pak1ip1 has been implicated in the regulation of both cytoskeletal regulation through the inhibition of the serine/threonine kinase Pak1 [15], [16] and ribosome biogenesis [19], [20]. The multiple and complex cellular functions Pak1ip1 may mediate, presents a challenge in understanding the molecular and cellular mechanisms underlying the median cleft in the *mray/mray* animals. To explain the midline cleft in homozygous mutants, we first considered local effects such as a specific deficit in patterning and specification of midfacial structures during development. During midgestational stages (E8.5 – E10) the frontonasal process, which will give rise to midfacial structures, is characterized by the expression of several factors that exert proliferative activity and are critical for proper outgrowth. Of particular relevance in this context are members of the fibroblast growth factor (Fgf) family and sonic hedgehog (Shh) [24]–[26]. Interestingly, Pak1 has been shown in a recent study to participate in the Fgf signaling pathway, which also interacts with the Shh pathway [27]. To examine possible changes in the expression of genes required to form the medial face we carried out whole-mount RNA *in situ* hybridization for *Fgf8*, *Fgf17*, and *Shh* on wild-type, heterozygotes, and mutants. Analyzing stages E9.0 to E11.5 (figure 5 and data not shown), we detected only mild changes in expression levels and distribution of these molecular markers but largely intact expression domains. Specifically, at E9.5 the midfacial expression domains of *Fgf8* and *Fgf17* largely overlapped with the frontonasal prominence and formed bilateral stripes around the midline, irrespective of genotype. Some low-level variation in the size of the expression domains, we attributed to size differences between wild-type and *mray/mray* embryos. *Shh* displayed a similar, but ventrally shifted, expression pattern, which was slightly diminished in the mutant compared to the wild-type but correlated well with the overall smaller size of the mutant embryos. Overall, our results suggested, if any, a minor contribution of midfacial molecular patterning to the manifestation of midline clefting in homozygous *mray* mutants.

### Subcellular localization of Pak1ip1 and Tp53 regulation

**Figure 6:**
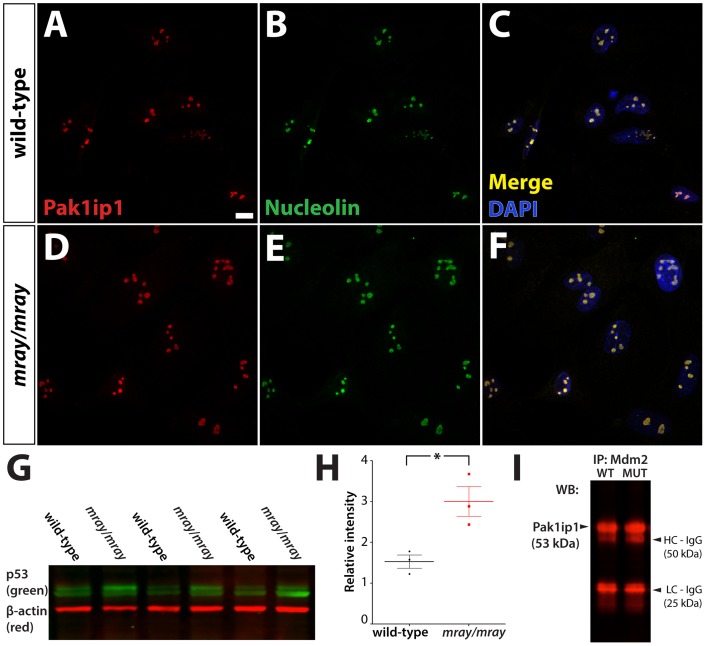
Pak1ip1 localizes to the nucleolus and its mutation increases Tp53 levels. Intracellular distribution of Pak1ip1 revealed through immunofluorescence in cultured primary fibroblasts derived from wild-type (**A–C**) and homozygous mutant embryo (**D–F**). Fluorescent labeling for Pak1ip1 (**A, D**), nucleolin (**B, E**), and merged colors with DAPI (**C, F**) show an exclusively nucleolar localization in wild-type (**A–C**) and mutant cells (**D–F**) perfectly overlapping with the nucleolin signal. Scale bar is 10 μm. The western blot analysis in **G** performed on total lysate form E10.5 embryos reveals an upregulation of Tp53 in *mray/mray* mutants over wild-type littermates. The graph in **H** shows the results of the quantitative and statistical analysis of relative band intensity compared to background for wild-type (black) and *mray/mray* (red) samples. Dots correspond to individual data points, the thick lines show the mean, and the thin bars the standard error within the sample sets. A Student's t-test was used to assign significance to the data; a p-value <0.05 was obtained from the analysis. In **I**, the results of a Mdm2 co-IP are shown, which was used to pull Pak1ip1 protein from E13.5 embryo lysates. No differential signal intensity can be detected between wild-type and homozygous mutants for the subsequent western analysis of Pak1ip1.

The phenotype of homozygous *mray* carriers has a complex and pleiotropic nature, which affects many organs and tissues without interfering with their initial development. In previous studies, Pak1ip1 has been shown to localize to the nucleolus and participate in ribosome biogenesis [19], [20]. Indeed, the overall reduction in size of homozygous *mray* mutants could be well explained by a general deficit in protein biosynthesis resulting in a prolonged cell cycle or even cell cycle arrest and reduced growth. To obtain further insight into the function of Pak1ip1 and its possible deregulation in the *mray* mutant, we examined its intracellular distribution in cells derived from wild-type and affected embryos. Primary cultures of fibroblasts obtained from E13.5 day old embryos were labeled with α-Pak1ip1 and α-nucleolin antibodies. Our results confirmed an exclusive localization of Pak1ip1 to the nucleolus, completely overlapping with nucleolin immunoreactivity (figure 6). Interestingly, we did not see any apparent differences in expression levels or subcellular distribution of Pak1ip1 between wild-type and mutant cells, suggesting that even though the *mray* allele disrupts the function of Pak1ip1 it does not affect its localization in the cell.

A recent study by Yu *et al*. identified a regulatory role for Pak1ip1 in cell growth and proliferation, via direct interaction with the tumor protein 53 (Tp53)-murine double minute 2 (Mdm2) loop upon detection of cellular stress [20]. Specifically, Pak1ip1 can either induce the Tp53-Mdm2 loop indirectly by activating the Mdm2-binding ribosomal proteins Rpl5 and Rpl11 that act as nucleolar stress signals or directly by binding and inhibiting the ability of Mdm2 to ubiquitinate Tp53. Consequently, knockdown of Pak1ip1 in cell culture has been shown to cause a decrease in proliferation by inducing Tp53 dependent G1 cell cycle arrest. In order to determine if the developmental defects seen in the *mray/mray* mice are indeed caused by deregulation of the Tp53 pathway, we performed a western blot analysis to compare Tp53 protein levels between wild-type and *mray/mray* embryos at E10.5 (Figure 6). We found Tp53 levels to be significantly twofold increased in the mutant further supporting the notion that the ribosomal function of Pak1ip1 and the associated upregulation of Tp53 in the homozygous *mray* mutants as being instrumental in the manifestation of the craniofacial anomalies we see in this line of mice.

To further uncover the mechanism by which mutant Pak1ip1 causes an upregulation in Tp53 levels, we examined its interaction with Mdm2 using co-immunoprecipitation (Co-IP) analysis. Total lysates from whole E13.5 embryos were immunoprecipitated using a Mdm2 antibody and probed with a Pak1ip1 antibody, which did not detect any differential binding between mutant (H122Y) and wild-type Pak1ip1 protein to Mdm2 (figure 6, I). This is in support of the notion that increased Tp53 levels in the *mray/mray* mutants are caused by aberrant ribosome biogenesis and increased nucleolar stress due to faulty or insufficient levels of translation. In summary, we conclude that the *mray* allele does neither interfere with the subcellular localization of Pak1ip1 nor with its ability to interact with Mdm2 but apparently causes defects in ribosome biogenesis that elicit a nucleolar stress response with associated Tp53 upregulation.

### Reduced proliferation rates of *mray/mray* cells can be rescued by Tp53 inhibition

**Figure 7:**
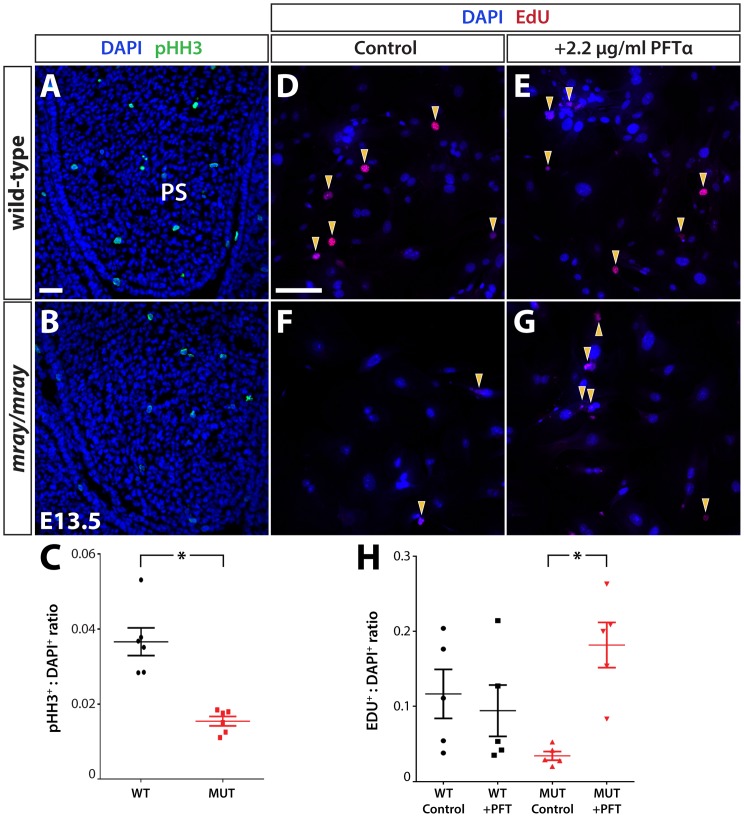
Proliferation deficits in *mray/mray* embryos can be rescued *in vitro* by Tp53 inhibition. Coronal sections through the palatal shelves of wild-type (**A**) and *mray/mray* mutant embryos (**B**) at E13.5 demonstrate a severe reduction in mitotic cells in the mutant as detected by pHH3 immunofluorescence. The quantification of the results is shown in **C** as a ratio of pHH3^+^ to DAPI^+^ cells. * represents a p-value <0.02. EdU cell proliferation assay on cultured primary facial fibroblasts from wild-type (**D**) and *manta-ray* homozygous mutants (**F**) reveals a reduced rate of proliferation in mutant cells. This deficit can be averted by treatment with the Tp53 inhibitor pifithrin-α, which significantly increased cell proliferation in mutant cells, but had no apparent effect on wild-type cells as shown in the quantification in **H** given as a ratio of EDU^+^ to DAPI^+^ cells. A total of five independent fields were analyzed between three slides for the experiment. EDU^+^: DAPI^+^ ratios for individual fields are represented by the circles (WT control), squares (WT + PFTα), triangles (MUT control) and upside down triangles (MUT + PFTα). Middle bar represents the mean value, with top and bottom bars representing standard error of the mean. * represents a p-value <0.02. EDU^+^: DAPI^+^ ratio was not significantly different between WT control, WT + PFTα, and MUT + PFTα groups. MUT: mutant, PS: palatal shelves, WT: wild-type. Scale bars are 30 μm.

Since Tp53 protein levels are elevated in mutant embryos with possible G1 cell cycle arrest, we sought to examine whether this leads also to a reduction in the rate of cell proliferation within developing craniofacial structures, as expected. We decided to count mitotic cells within the palatal shelves at stage E13.5 prior to their elevation since they present confined structures facilitating sampling. Consistent with our hypothesis, the palatal shelves of homozygous *mray* mutant embryos show a significant decrease in the ratio of phosphorylated histone-H3 immunoreactive cells compared to the palatal shelves of wild-type animals (figure 7, A–C). Since this loss of proliferative activity in mutant cells is likely caused by higher Tp53 levels, we examined whether Tp53 inhibition has the capacity to rescue the decreased proliferation rate. To that effect, we isolated facial fibroblasts from E13.5 wild-type and mutant embryos and cultured them with or without the Tp53-specific inhibitor pifithrin-α (PFT-α). Proliferation rates were assessed by Click-iT EdU cell proliferation assays utilizing incorporation and detection of the modified nucleoside, EdU (5-ethynyl-2′-deoxyuridine) in dividing cells. Comparing the ratio of dividing over non-dividing cells within a two-hour interval, we identified a substantial reduction in mitotic cells in the mutant (figure 7, D–H). Administration of 2.2 mg/ml of PFT-α in the culture media of wild-type and mutant cells lead to a significant increase in the proliferation rate of mutant cells but had no significant effect on wild-type cells compared to controls. Overall, inhibition of Tp53 using PFT-α increased proliferation levels of *mray/mray* cells to levels comparable to their wild-type counterparts.

### *PAK1IP1* association and sequencing studies

**Figure 8:**
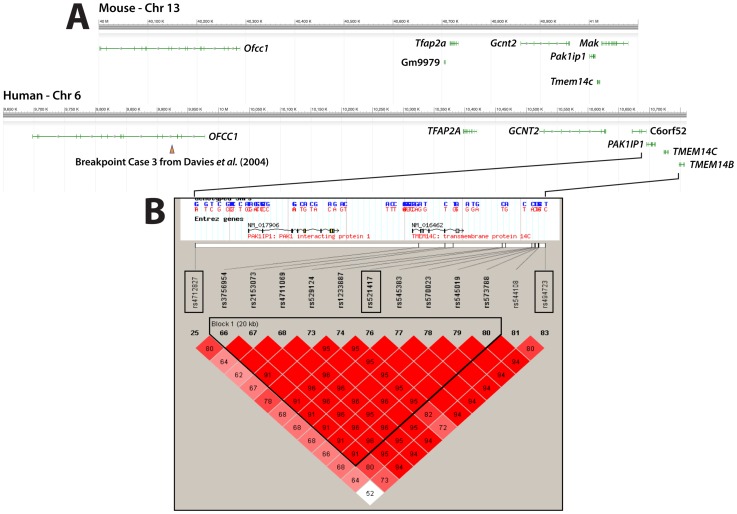
Mouse/Human synteny and Haploview output showing linkage disequilibrium relationships of the analyzed SNPs. Approximately 1 Mb of syntenic aspects of mouse chromosome 13 and human chromosome 6 containing the *Pak1ip1/PAK1IP1* genes are shown in National Center for Biotechnology Information Sequence Viewer 2.25 generated images in **A**. Positions and exon/intron structure of genes are indicated as green lines. In addition, the translocation breakpoint closest to *PAK1IP1* reported in [8] is indicated through an arrowhead. In **B** the Haploview generated LD block structure encompassing 100 kb around *PAK1IP1* for the Han Chinese and Japanese populations combined is presented. The upper part shows the genomic region of the *PAK1IP1* gene and boxed the SNPs used in our association analysis and their physical position relative to *PAK1IP1*. Marker to marker D′ statistics are shown below with linkage disequilibrium (LD) blocks defined as solid spine of LD with a D′>0.8. Cells with no value indicate D′ = 1.0. Color intensity indicate strength of association, the darker the cell the greater the LD between individual SNPs. The haplotype block is outlined.

Next we examined the possibility that *PAK1IP1* (6p24.2) may be a human disease gene as was suggested by studies that mapped translocation breaking points associated with cleft lip and palate to human chromosome 6p24.1 [7], [8]. *PAK1IP1* maps at a distance of less than 1 to these breaking points hinting at a possible role as disease gene or contributing factor to the manifestation of the craniofacial pathology in these patients (figure 8). To examine this possibility, we conducted sequencing and association studies on a set of samples obtained from 271 Filipino non-syndromic cleft lip and palate patients and their respective parents. First, we established a Hapmap haplotype block for the *PAK1IP1* region looking for SNPs with a minor allele frequency (MAF) of at least 0.2 in the Chinese (CHB) and Japanese (JPT) populations. *PAK1IP1* spans 14.78 kb and does not contain SNPs with a MAF higher than 0.15 for these populations. We then proceeded to expand the search for informative SNPs to 100 kb around the gene and selected 3 SNPs to genotype: rs4712827, rs521417, and rs494723, marked with boxes in the plot in figure 8. Our association analysis showed that marker rs494723 was significantly associated with CL/P cases (p = 0.01; tables 1 and 2). A genome-wide association study reported a SNP about 1 Mb from *PAK1IP1* that reached a p value of 5.50 E-05 (rs760788) [3]. In addition, we conducted a case-control analysis in 259 Polish cases and 493 unrelated Polish controls. Results are listed in tables 3 and 4; similarly to our Filipino trio association results only rs494723 showed borderline significance (p = 0.0437).

**Table 1:** Family association results.

Marker	Allele	afreq	Fam#	Z	P
rs521417	C	0.748	105	1.635	0.102081
rs4712827	T	0.627	151	1.082	0.279393
**rs494723**	**T**	**0.695**	**108**	**2.562**	**0.010406**

Results of the family-based association test on Filipino trios consisting of a nonsyndromic CL/P patient and both parents.

**Table 2:** Results of the haplotype analysis of the selected SNPs in the family-based association study.

Haplotype	Frequency	Fam#	Z	P
**CTT**	**0.367**	**92.4**	**2.028**	**0.042519**
**CCC**	**0.235**	**80**	**−1.912**	**0.055844**
ATT	0.166	67.4	**−**0.755	0.450089
CCT	0.082	41.9	1.187	0.235181
ACC	0.07	32.3	**−**0.939	0.347959
CTC	0.047	25.1	**−**0.726	0.467776
ACT	0.019	9.6	N/A	N/A
ATC	0.014	7.8	N/A	N/A

Results of the haplotype analysis of markers rs521417, rs4712827, and rs494723 for the Filipino trios. afreq: allele frequency, Fam#: number of informative families, Z: z-score, P: p-value.

**Table 3:** Case-control association results.

Marker	Alleles^a^	MAF^b^	Genotypes cases^c^	Genotypes controls^c^	P_genotypic_ value	P_allelic_ value	P_trend_ value	OR_dominant_ (95% CI)^d^; p-value	OR_recessive_ (95% CI)^e^; p-value
rs521417	a/C	0.38	111/112/36	182/244/67	0.2287	0.2835	0.2808	0.780 (0.54 – 1.060); 0.1125	1.026 (0.664 – 1.588); 0.9067
rs4712827	c/T	0.46	78/118/63	143/247/103	0.4277	0.6679	0.6716	0.948 (0.682 – 1.318); 0.7509	1.217 (0.851 – 1.740); 0.2809
rs494723	c/T	0.29	151/93/15	249/206/38	0.1165	**0.0437**	**0.0426**	**0.730 (0.539−0.989);** **0.0418**	0.736 (0.397 – 1.365); 0.3292

Results of the Polish case-control association analysis. ^a^Uppercase denotes the more frequent allele in the control samples, ^b^MAF, minor allele frequency calculated from the control samples, ^c^The order of genotypes: DD/Dd/dd (d is the minor allele), ^d^Dominant model: dd + Dd vs DD (d is the minor allele), ^e^Recessive model: dd vs Dd + DD (d is the minor allele).

**Table 4:** Results of the haplotype analysis of the selected SNPs in the case-control study.

SNP Combination	X^2^	Global p-value
**2-marker window**		
rs4712827_rs521417	1.613	0.656
rs521417_rs494723	3.967	0.265
**3-marker window**		
rs4712827_rs521417_rs494723	9.404	0.152

Results of the haplotype analysis of markers rs521417, rs4712827, rs494723 in the Polish unrelated non-syndromic CL/P cases. Empirical 5% quantile of the best p-value: 0.009972 (1,000 permutations).

We proceeded to sequence the 10 exons of *PAK1P1* in samples obtained from 271 Filipino non-syndromic cleft lip and palate patients. We identified 3 not previously reported noncoding variants, an A/C change at chr6:10,695,513 was found in 4 patients while chr6:10,707,487 (A/T) and chr6:10,707,787 (A/G) was only found in one patient each. These variants are considered new since they are not present in public databases, which include over 7,000 individually sequenced whole exomes [28], [29]. However, no unambiguously deleterious mutations were identified with this approach.

## Discussion

The Centers for Disease Control estimate that orofacial clefts with an average worldwide rate of 1 to 700 are the most common birth defects. Consequently, the search for genetic factors causing these defects is a high priority in addressing diagnostic, preventive, and therapeutic needs for orofacial clefts [30]. In a forward genetic screen in mice, we identified a mutation in the *Pak1ip1* gene that causes a median cleft with cleft palate in homozygous mutants. These animals present us with the first described mutant allele of the *Pak1ip1* gene in mice. Pak1ip1 is a molecule of approximately 50 kDa almost entirely comprised of five WD40 repeats, which have been characterized as sites of protein-protein interaction to which proteins can bind either stably or reversibly to form larger assemblies. The structural characteristics of Pak1ip1 indicate that it has a multitude of distinct molecular interactions underlining its complex role in cellular function. Originally, it was described as a negative regulator of the serine-threonine kinase Pak1 (P21-activated kinase) thus mediating processes of gene expression, cytoskeletal actin and tubulin assembly, cell cycle control, and apoptosis [15], [16]. Subsequent studies have shown that next to its presumed function in cytoskeletal regulation, Pak1ip1 also localizes to the nucleolus where it is involved in ribosome biogenesis, specifically the assembly of the 60S ribosomal subunit [19]. As a preribosomal factor, Pak1ip1 facilitates ribosomal RNA processing and plays a regulatory role in cell growth and proliferation, apparently through direct interaction with the tumor protein 53 (Tp53)-murine double minute 2 (Mdm2) loop upon cellular stress [20]. Its function suggests impairment in ribosome biogenesis as the underlying cause of the observed craniofacial defects in *Pak1ip1^mray/mray^* mutants, which is also supported by our findings. Specifically, we identified both an exclusive localization of Pak1ip1 to the nucleolus and an upregulation of Tp53 levels in homozygous *mray* embryos during the early formation of craniofacial structures (figure 6), consistent with the notion that cellular stress causes Tp53-dependent G1 cell-cycle arrest associated with the reduced outgrowth of midfacial structures. Furthermore, we found the rate of proliferation within the developing palatal shelves of the mutants to be significantly decreased and pharmacological inhibition of Tp53 with pifithrin-α in primary facial fibroblasts was able to rescue this proliferative deficit *in vitro*. Interestingly, the *mray* allele neither interferes with the nucleolar localization of mutant Pak1ip1^H122Y^, which is expected since its nuclear and nucleolar localization signals were previously mapped to the C-terminus of the protein (corresponding to amino acid residues 366–382 in mouse) [20], nor with its affinity to the Tp53 regulator Mdm2. Yu *et al*. proposed two mechanisms by which Pakip1 can elicit an upregulation of Tp53 levels resulting in cell cycle arrest. First, by overexpression and direct inhibition of Mdm2 inhibiting its ability to ubiquitinate and degrade Tp53 and second, indirectly after knockdown, by increasing the concentration of free ribosomal proteins L5 and L11, which also bind to and inhibit Mdm2. In Mdm2-Pak1ip1 co-immunoprecipitation experiments using either wild-type or mutant lysates, we did not detect any differences between genotypes strongly suggesting the *mray* allele as a loss-of-function mutation disabling the proper participation of the mutant protein in ribosome biogenesis. We also examined a possible local, midfacial deregulation of signaling molecules important for midfacial outgrowth such as *Fgf8*, *Fgf17*, and *Shh,* but detected only mild changes in the craniofacial expression of these genes. It is very unlikely that low-level changes in the midfacial expression of these genes causes the midline defects seen in homozygous *mray* mutants, particularly in light of a recent study by Griffin *et al.*, which found that a loss of approximately 80% of *Fgf8* expression in *Fgf8^null/neo^* mice is required to cause a midfacial cleft of comparable extent to the cleft in the *Pak1ip1^mray/mray^* mutants [31]. Overall, our findings are consistent with the hypothesis that aberrant ribosome biogenesis associated with cell cycle arrest caused by increased Tp53 activity is the major mechanism underlying the craniofacial hypoplasia displayed by homozygous *mray* mutants.

Pathological changes of a similar nature to the *mray/mray* phenotype can be seen in human ribosomopathies such as Diamond-Blackfan anemia (DBA) or Treacher Collins syndrome (TCS), which are associated with craniofacial malformations including cleft palate [32], [33]. Further, in a recent study in *Tail short* (*Ts*) mice, which are characterized by a partially penetrant midfacial cleft of very similar appearance to the phenotype seen in homozygous *mray* mutants, causative mutations were identified in the gene encoding the ribosomal protein L38 (Rpl38) [34]. Interestingly, in mice carrying mutations in the Tcof gene, a successful rescue of the mutant craniofacial phenotype was achieved by both pharmacological and genetic inhibition of Tp53. Taken together with the results we present here, we suspect that in future studies a similar rescue may be possible for the *mray/mray* mutant mice too.

While orofacial clefts are very common birth defects, median clefts such as the one displayed by the *Pak1ip1^mray/mray^* animals are extremely rare. Median clefts arise from the failure of the frontonasal processes to fuse along the midline during development. To this date no causative mutation for isolated midline clefting in humans has been identified but midline clefts are commonly seen in holoprosencephaly syndromes for which several genes have been identified. In addition, a few genetically defined mouse models exist. These include compound mutants of the (*Alx4* **^−^** ^*/*−^ */Cart1^+/^* ^−^) [35], retinoic acid receptors and α and γ double mutants (*Rarα* **^−^** ^*/*−^ */Rarγ* **^−^** ^*/*−^) [36], and platelet-derived growth factor receptor mutants (*Pdgfrα^ph/ph^, Pdgfrα* **^−^** ^*/*−^) [37], [38]. In *Tail short* (*Ts*) mice, which show with partial penetrance a midfacial cleft, causative mutations were identified in the gene encoding the ribosomal protein L38 (Rpl38) [34]. This finding may point to a more general role for ribosome biogenesis defects and associated deficits in protein biosynthesis as an underlying cause for median clefts.

The possible role for *PAK1IP1* (6p24.2) in human disease was suggested by studies mapping translocation breaking points associated with cleft lip and palate in affected individuals to human chromosome 6p24.1 [7], [8]. *PAK1IP1* maps between 0.5 and 1 Mb of these breaking points making it a possible candidate or contributing factor to the manifestation of the disease phenotype in these patients. To examine this possibility, we conducted sequencing and association studies. While our results did not provide clear evidence associating *PAK1P1* with orofacial clefts, some association was seen for the nearby marker rs494723 and for a haplotype (Table 1).

Several factors may explain the fact that we did not find a mutation within the coding sequences of *PAK1IP1*. First, as for the *mray* allele, prenatal lethality of human carriers may eliminate them from the general and our sample population. Second, none of the samples tested was derived from a patient with the specific pathological features seen in the *Pak1ip1^mray/mray^* mutants, a median orofacial cleft with cleft palate. Third, we only sequenced the *PAK1IP1* coding region in the Filipino samples since Schultz *et al.* have demonstrated linkage to a neighboring region (6p23) in this population [11]; however,it remains possible that etiologic variants are harbored in non-coding regulatory elements. Future studies can utilize different sample sets, particularly sets that include samples from patients with median clefts and relevant neuronal defects as well as populations of different genetic background. In summary, our findings point towards an important role for Pak1ip1 as a developmental regulator, particularly in the formation of the cranial skeleton, but require further study to understand its disease relevance in the manifestation of congenital craniofacial malformations.

## Materials and Methods

### Ethics statement

The study was approved by the University of Iowa Institutional Review Board (IRB) in the United States under IRB-01 ID #199804080; Title: Studies of Genes Involved in Human Head, Face and Eye Disorders. Informed written consent was obtained from all participating individuals or legal caretakers and the data were analyzed anonymously. Filipino cases were recruited through ongoing follow-ups of families originally identified during Operation Smile medical missions and all practices were approved by the Internal Review Board of the Corazon Locsin Montelibano Memorial Regional Hospital [39]. Polish case eligibility was ascertained by clinicians using detailed diagnostic information from medical records and following guidelines established by the Medical Ethics Committee of the Supreme Medical Council of the Polish Chamber of Physicians and Dentists (PCHPD). The study was approved by the ethical committee of the Poznan University of Medical Sciences. Written and oral consent was obtained from all participants. Mice were housed in specific-pathogen-free facilities approved by the Association for Assessment and Accreditation of Laboratory Animal Care International (AALAC). All animals were handled in accordance with protocols approved by the University of California at Davis Institutional Animal Care and Use Committee.

### Animal husbandry and genotyping

The colony of animals carrying the *Pak1ip1^mray^* allele (induced on C57BL/6J background) is maintained by crossing male carriers with FVB/NJ females. This mode of outcross is currently in the ninth generation without any changes in penetrance or variability of the mutant phenotype. All embryos presented in the phenotypic analysis of this study were produced from carriers crossed for at least four generations onto an FVB/NJ background. Routine genotyping was performed by amplifying microsatellites D13Mit63 and D13Mit177.

### Positional mapping and candidate gene sequencing

The *mray* line was recovered in a genetic screen for recessive mutations disrupting forebrain development [23]. Positional mapping was performed using simple-sequence length polymorphic markers available from the Whitehead Institute for Biomedical Research, MIT. Initial linkage was established after a genome-wide scan using 82 simple sequence repeat markers on 12 DNA samples from both carriers and mutant embryos. For high-resolution meiotic mapping, we scored separate crosses resulting in over 1572 generated meioses (carriers and embryos counted). Using this approach, we located the *mray* mutation to an interval on chromosome 13 between markers D13Mit177 (38.975 Mb) and D13Mit63 (42.709 Mb) in the NCBI mouse assembly (Build 37.2, current assembly). The identified genetic interval contained 22 protein-coding genes of which all exons were amplified and sequenced from genomic DNA samples. For every set of primers, we used samples of the two parental strains (C57BL/6J and FVB/NJ) and two samples of affected mutants. All primer sequences are available upon request. Sequence comparisons were carried out using Mutation Surveyor® DNA variant analysis software (SoftGenetics, LLC, State College, PA) and BLAST (National Center for Biotechnology Information). Sequences of insufficient quality or read length were dismissed from any comparisons and repeated with revised primer sets. To further confirm that the mutation in *Pak1ip1* is causative, we genotyped a total of 116 phenotypically mutant embryos (232 meioses) of stages E11.5 to E14.5, which are easy to score. For genotype analysis, we used a restriction length polymorphism created by the *manta-ray* allele (additional *Hind*III site) and found that all affected embryos carried the mutant variant in a homozygous state.

### Embryo imaging and histology

Embryos of all developmental stages analyzed, were recovered after timed pregnancies and fixed by immersion in 4% paraformaldehyde in phosphate buffered saline (PBS). For some whole-mount imaging, embryos were stained with ethydium bromide at 10 μg/ml in PBS for several hours, briefly washed with several exchanges of PBS, and photographed under fluorescent illumination. Nissl stained (cresyl violet) or hematoxylin and eosin stained histological sections of 10 μm thickness were prepared from paraffin-embedded tissue using standard procedures. All RNA *in situ* hybridization on whole-mount embryos and sections was performed using standard procedures as previously described [40]. For each marker and developmental stage shown, we analyzed per experiment at least three embryos of each genotype (wild-type, heterozygote, mutant) and carried out every experiment at least twice. The *Pak1ip1* riboprobe was transcribed from a 1.1 kb cDNA fragment containing the entire coding region of the gene (base pairs 321–1469 in NM_026550). Plasmids to transcribe riboprobes for *Fgf8*, *Fgf17*, and *Shh* were kindly provided by Dr. John L.R. Rubenstein.

### Immunocytochemistry and immunohistochemistry

Primary fibroblasts were isolated from the facial mesenchyme of E13.5 embryos. Upon removal from the uterus, embryos were placed into ice-cold HBSS where the facial mesenchymal tissue was then isolated. The tissue was treated with DNase and trypsin-EDTA for 13 minutes at 37°C, the dissociated cells resuspended in Dulbecco's Modified Eagle Medium (DMEM), and plated on poly-D-lysine coated Lab-Tek chamber slides (Nunc, Rochester, NY). After incubation for at least 24 h cells were fixed in ice cold 4% PFA in PBS (pH 7.4) for 15 minutes prior to being permeabilized using PBS +0.25% Triton X-100. After blocking in 1% BSA (in 1× PBS +0.1% Tween-20) for one hour the cells were incubated in primary antibodies for Pak1ip1 (1∶400; Orbigen, San Diego, CA) and nucleolin (1∶1000; AbCam, Cambridge, MA). Cells were washed with PBS and incubated with the appropriate secondary antibodies (CY3, 1∶250 and 488M 1∶1000) in 1% BSA in PBS (Alexa Fluor, Invitrogen, Carlsbad, CA). Prior to the final PBS rinse, cells were counterstained with the fluorescent nucleic acid stain 4',6-diamidino-2-phenylindole (DAPI, Invitrogen, Carlsbad, CA), coverslipped, and imaged using a Nikon scanning confocal laser microscope with the associated proprietary software (Nikon Instruments, Tokyo). Phospho-histone H3 immunofluorescent analysis was carried out on 14 mounted sections cut from tissue fixed in 4% paraformaldehyde/PBS and cryoprotectively frozen in 30% sucrose using an α-pHH3 antibody (1∶400, Abcam, Cambridge, MA). Same buffers and incubations were used as described above.

### Western analysis

25 μg of protein lysates obtained from whole E10.5 embryos were run on a Nu-Page 4–12% Bis-Tris gels (Invitrogen, Carlsbad, CA). Proteins were transferred to nitrocellulose membranes and blocked in Odyssey blocking buffer (Li-Cor Biosciences, Lincoln, NE). Membranes were incubated with Tp53 primary antibody (1∶,000; Cell Signaling Technology, Boston, MA) diluted in Odyssey blocking buffer +0.1% Tween-20, overnight at 4°C. After washing, we incubated with Li-Cor IR secondary antibody (1∶15,000; Li-Cor Biosciences, Lincoln, NE) in Odyssey blocking buffer +0.1% Tween-20, washed, and imaged on the Li-Cor Odyssey IR scanner. The process was repeated with β-actin as loading control (Novus Biologicals, Littleton, CO). Protein sizes were assigned using the Li-Cor infrared two-color protein marker. Signal intensity relative to background was determined using Li-Cor Image Studio version 2.0 software and Prism version 6 software was used to calculate standard error and perform a t-test in order to assign statistical significance to the results (GraphPad Software, La Jolla, CA).

### Co-immunoprecipitation analysis

All experiments were conducted in triplicate using components of the Immunoprecipitation Kit – Dynabeads Protein G from Invitrogen (Carlsbad, CA). 1 mg of whole embryo (E13.5) protein lysate from wild-type and *mray/mray* animals was incubated with 10 μg of Mdm2 antibody (Abcam, Cambridge, MA) and 200 μl of antibody binding buffer overnight on a rocker at 4°C. Subsequently, 50 μl of protein-G dynabeads were added to the mixture, and incubated on a rocker at 4°C for 2.5 hours. Following this step, the bead-antibody-protein complex was washed thrice with 200 μl of wash buffer before being suspended in 30 μl of a 70% 4× LDS and 30% 10× reducing agent solution. The samples were then boiled for 10 minutes at 70°C prior to being loaded (15 ul) onto a 4–12% bis-tris gel (Invitrogen). Western analysis was carried out as described above exposing the membrane to 1 μg/ml of Pak1ip1 antibody (Orbigen, San Diego, CA) for 12 hours at 4°C.

### EdU proliferation assay

Primary facial fibroblast cells from wild-type and homozygous *manta-ray* mutants were cultured in DMEM growth media on 4-chambered culture slides (Nunc, Rochester, NY) for 24 hours with or without 2.2 μg/ml of the Tp53 inhibitor pifithrin-α (Sigma) and then exposed to 10 μM 5-ethynyl-2'-deoxyuridine (EdU, Invitrogen, Carlsbad, CA) for two hours at 37°C. Subsequently, cells were fixed in 4% paraformaldehyde in PBS prior to click chemistry development of EdU and DAPI staining. For the quantification of EDU^+^ and DAPI^+^ cells, a total of five randomly selected independent fields (600×600 μm) between three slides for each experimental group were analyzed. Each field contained approximately 100 cells. Statistical significance was assigned using a two-tailed Student's t-test with Prism version 6 software (GraphPad Software, La Jolla, CA).

### Human subjects

Two hundred and fifty nine NSCL/P patients (228 cases with cleft lip and palate and 31 cases with cleft lip only) were identified from the Department of Pediatrics and Pediatric Surgery at the Institute of Mother and Child in Warsaw as well as from the University Clinic of the Medical Academy in Wroclaw and the Department of Plastic Surgery Specialist Medical Center in Polanica Zdroj. The control group was composed of 493 healthy individuals with no family history of clefts or other major congenital anomalies. Controls were matched with the NSCL/P patients for age, sex and the place of residence. Peripheral blood samples were obtained from selected individuals in both populations and DNA was extracted.

### Association study

Single nucleotide polymorphisms (SNPs) were selected using the Hapmap Project and Haploview tagged SNPs for the Chinese and Japanese population, restricting minor allele frequencies to 0.2 and selecting enough SNPs to provide at least 80% genetic coverage of haplotype blocks [41]. Selected SNPs (Table 1) were genotyped by allelic discrimination using Taqman assays (Applied Biosystems, Foster City, CA, USA) in 307 Filipino trios consisting of a nonsyndromic CL/P patient and both parents. Association was assessed using Familiy Based Association Test (FBAT) [42].

### Case-control Study

The same SNPs as in the association study were genotyped by high-resolution melting curve analysis (HRM) on the *LightCycler* 480 system (Roche Diagnostics, Mannheim, Germany) in a set of 259 nonsyndromic CL/P cases and 493 unaffected controls. The differences in allele and genotype frequencies between case and control mothers were determined using Chi-square test. SNPs were tested for association with NSCL/P using the Cochran-Armitage trend test. The strength of association was estimated by Odds Ratio (OR) and a corresponding 95% confidence interval (95%CI). Both recessive and dominant inheritance models were analysed. A p-value < 0.05 was considered statistically significant. Haplotype based association analysis was performed using the UNPHASED 3.1.5 program with the following analysis options: all window sizes, full model and uncertain haplotype option. Haplotypes with a frequency below 0.01 were set to zero [43]. Significant p-values were corrected using the 1000-fold permutation test.

### Direct sequencing

The *PAK1IP1* coding region was sequenced in 273 Filipino nonsyndromic CL/P patients. Primers were designed using primer3 and are available upon request [44]. Templates for sequencing were generated by PCR in an Applied Biosystems Gene Amp PCR System 9700, as described in Mansilla *et al*. [45]. Sequencing reactions were resolved on an ABI Prism 3700 analyzer and analyzed by Polyphred 4.0 and Consed.

## References

[1] Murray JC (2002) Gene/environment causes of cleft lip and/or palate. Clin Genet 61: 248–256. 12030886 10.1034/j.1399-0004.2002.610402.x

[2] Dixon MJ, Marazita ML, Beaty TH, Murray JC (2011) Cleft lip and palate: understanding genetic and environmental influences. Nat Rev Genet 12: 167–178. 21331089 10.1038/nrg2933 PMC3086810

[3] Beaty TH, Murray JC, Marazita ML, Munger RG, Ruczinski I, et al (2010) A genome-wide association study of cleft lip with and without cleft palate identifies risk variants near MAFB and ABCA4. Nat Genet 42: 525–529. 20436469 10.1038/ng.580 PMC2941216

[4] Birnbaum S, Ludwig KU, Reutter H, Herms S, Steffens M, et al (2009) Key susceptibility locus for nonsyndromic cleft lip with or without cleft palate on chromosome 8q24. Nat Genet 41: 473–477. 19270707 10.1038/ng.333

[5] Mangold E, Ludwig KU, Birnbaum S, Baluardo C, Ferrian M, et al (2010) Genome-wide association study identifies two susceptibility loci for nonsyndromic cleft lip with or without cleft palate. Nat Genet 42: 24–26. 20023658 10.1038/ng.506

[6] Jugessur A, Murray JC (2005) Orofacial clefting: recent insights into a complex trait. Curr Opin Genet Dev 15: 270–278. 15917202 10.1016/j.gde.2005.03.003 PMC2442458

[7] Davies AF, Stephens RJ, Olavesen MG, Heather L, Dixon MJ, et al (1995) Evidence of a locus for orofacial clefting on human chromosome 6p24 and STS content map of the region. Hum Mol Genet 4: 121–128. 7711723 10.1093/hmg/4.1.121

[8] Davies SJ, Wise C, Venkatesh B, Mirza G, Jefferson A, et al (2004) Mapping of three translocation breakpoints associated with orofacial clefting within 6p24 and identification of new transcripts within the region. Cytogenet Genome Res 105: 47–53. 15218257 10.1159/000078008

[9] Milunsky JM, Maher TA, Zhao G, Roberts AE, Stalker HJ, et al (2008) TFAP2A mutations result in branchio-oculo-facial syndrome. Am J Hum Genet 82: 1171–1177. 18423521 10.1016/j.ajhg.2008.03.005 PMC2427243

[10] Rahimov F, Marazita ML, Visel A, Cooper ME, Hitchler MJ, et al (2008) Disruption of an AP-2alpha binding site in an IRF6 enhancer is associated with cleft lip. Nat Genet 40: 1341–1347. 18836445 10.1038/ng.242 PMC2691688

[11] Schultz RE, Cooper ME, Daack-Hirsch S, Shi M, Nepomucena B, et al (2004) Targeted scan of fifteen regions for nonsyndromic cleft lip and palate in Filipino families. Am J Med Genet A 125A: 17–22. 14755461 10.1002/ajmg.a.20424

[12] Scapoli L, Pezzetti F, Carinci F, Martinelli M, Carinci P, et al (1997) Evidence of linkage to 6p23 and genetic heterogeneity in nonsyndromic cleft lip with or without cleft palate. Genomics 43: 216–220. 9244439 10.1006/geno.1997.4798

[13] Prescott N, Lees M, Winter R, Malcolm S (2000) Identification of susceptibility loci for nonsyndromic cleft lip with or without cleft palate in a two stage genome scan of affected sib-pairs. hum genet 106: 345–350. 10798365 10.1007/s004390051048

[14] Moreno LM, Arcos-Burgos M, Marazita ML, Krahn K, Maher BS, et al (2004) Genetic analysis of candidate loci in non-syndromic cleft lip families from Antioquia-Colombia and Ohio. Am J Med Genet A 125A: 135–144. 14981713 10.1002/ajmg.a.20425

[15] Kim HW, Yang P, Qyang Y, Lai H, Du H, et al (2001) Genetic and molecular characterization of Skb15, a highly conserved inhibitor of the fission yeast PAK, Shk1. Mol Cell 7: 1095–1101. 11389855 10.1016/s1097-2765(01)00248-9

[16] Xia C, Ma W, Stafford LJ, Marcus S, Xiong WC, et al (2001) Regulation of the p21-activated kinase (PAK) by a human Gbeta -like WD-repeat protein, hPIP1. Proc Natl Acad Sci U S A 98: 6174–6179. 11371639 10.1073/pnas.101137298 PMC33441

[17] Parrini MC, Matsuda M, de Gunzburg J (2005) Spatiotemporal regulation of the Pak1 kinase. Biochem Soc Trans 33: 646–648. 16042564 10.1042/BST0330646

[18] Hofmann C, Shepelev M, Chernoff J (2004) The genetics of Pak. J Cell Sci 117: 4343–4354. 15331659 10.1242/jcs.01392

[19] Saveanu C, Rousselle JC, Lenormand P, Namane A, Jacquier A, et al (2007) The p21-activated protein kinase inhibitor Skb15 and its budding yeast homologue are 60S ribosome assembly factors. Mol Cell Biol 27: 2897–2909. 17308036 10.1128/MCB.00064-07 PMC1899936

[20] Yu W, Qiu Z, Gao N, Wang L, Cui H, et al (2011) PAK1IP1, a ribosomal stress-induced nucleolar protein, regulates cell proliferation via the p53-MDM2 loop. Nucleic Acids Res 39: 2234–2248. 21097889 10.1093/nar/gkq1117 PMC3064775

[21] Draptchinskaia N, Gustavsson P, Andersson B, Pettersson M, Willig TN, et al (1999) The gene encoding ribosomal protein S19 is mutated in Diamond-Blackfan anaemia. Nat Genet 21: 169–175. 9988267 10.1038/5951

[22] Gazda HT, Grabowska A, Merida-Long LB, Latawiec E, Schneider HE, et al (2006) Ribosomal protein S24 gene is mutated in Diamond-Blackfan anemia. Am J Hum Genet 79: 1110–1118. 17186470 10.1086/510020 PMC1698708

[23] Zarbalis K, May SR, Shen Y, Ekker M, Rubenstein JL, et al (2004) A focused and efficient genetic screening strategy in the mouse: identification of mutations that disrupt cortical development. PLoS Biol 2: E219. 15314648 10.1371/journal.pbio.0020219 PMC509294

[24] Bachler M, Neubuser A (2001) Expression of members of the Fgf family and their receptors during midfacial development. Mech Dev 100: 313–316. 11165488 10.1016/s0925-4773(00)00518-9

[25] Wang Y, Song L, Zhou CJ (2011) The canonical Wnt/beta-catenin signaling pathway regulates Fgf signaling for early facial development. Dev Biol 349: 250–260. 21070765 10.1016/j.ydbio.2010.11.004

[26] Hu D, Marcucio RS (2009) A SHH-responsive signaling center in the forebrain regulates craniofacial morphogenesis via the facial ectoderm. Development 136: 107–116. 19036802 10.1242/dev.026583 PMC2685963

[27] Jean S, Tremblay MG, Herdman C, Guillou F, Moss T (2012) The endocytic adapter E-Syt2 recruits the p21 GTPase activated kinase PAK1 to mediate actin dynamics and FGF signalling. Biol Open 1: 731–738. 23213466 10.1242/bio.2012968 PMC3507230

[28] The 1000 Genomes Project Consortium (2010) A map of human genome variation from population-scale sequencing. Nature 467: 1061–1073. 20981092 10.1038/nature09534 PMC3042601

[29] Kent WJ, Sugnet CW, Furey TS, Roskin KM, Pringle TH, et al (2002) The human genome browser at UCSC. Genome Res 12: 996–1006. 12045153 10.1101/gr.229102 PMC186604

[30] Lidral AC, Murray JC (2004) Genetic approaches to identify disease genes for birth defects with cleft lip/palate as a model. Birth Defects Res A Clin Mol Teratol 70: 893–901. 15578714 10.1002/bdra.20096

[31] Griffin JN, Compagnucci C, Hu D, Fish J, Klein O, et al (2013) Fgf8 dosage determines midfacial integration and polarity within the nasal and optic capsules. Dev Biol 374: 185–197. 23201021 10.1016/j.ydbio.2012.11.014 PMC4086262

[32] Vlachos A, Ball S, Dahl N, Alter BP, Sheth S, et al (2008) Diagnosing and treating Diamond Blackfan anaemia: results of an international clinical consensus conference. Br J Haematol 142: 859–876. 18671700 10.1111/j.1365-2141.2008.07269.x PMC2654478

[33] Marszalek B, Wojcicki P, Kobus K, Trzeciak WH (2002) Clinical features, treatment and genetic background of Treacher Collins syndrome. J Appl Genet 43: 223–233. 12080178

[34] Kondrashov N, Pusic A, Stumpf CR, Shimizu K, Hsieh AC, et al (2011) Ribosome-mediated specificity in Hox mRNA translation and vertebrate tissue patterning. Cell 145: 383–397. 21529712 10.1016/j.cell.2011.03.028 PMC4445650

[35] Qu S, Tucker SC, Zhao Q, deCrombrugghe B, Wisdom R (1999) Physical and genetic interactions between Alx4 and Cart1. Development 126: 359–369. 9847249 10.1242/dev.126.2.359

[36] Lohnes D, Mark M, Mendelsohn C, Dolle P, Dierich A, et al (1994) Function of the retinoic acid receptors (RARs) during development (I). Craniofacial and skeletal abnormalities in RAR double mutants. Development 120: 2723–2748. 7607067 10.1242/dev.120.10.2723

[37] Morrison-Graham K, Schatteman GC, Bork T, Bowen-Pope DF, Weston JA (1992) A PDGF receptor mutation in the mouse (Patch) perturbs the development of a non-neuronal subset of neural crest-derived cells. Development 115: 133–142. 1638976 10.1242/dev.115.1.133

[38] Soriano P (1997) The PDGF alpha receptor is required for neural crest cell development and for normal patterning of the somites. Development 124: 2691–2700. 9226440 10.1242/dev.124.14.2691

[39] Murray JC, Daack-Hirsch S, Buetow KH, Munger R, Espina L, et al (1997) Clinical and epidemiologic studies of cleft lip and palate in the Philippines. Cleft Palate Craniofac J 34: 7–10. 9003905 10.1597/1545-1569_1997_034_0007_caesoc_2.3.co_2

[40] Zarbalis K, Wurst W (2000) Expression domains of murine ephrin-A5 in the pituitary and hypothalamus. Mech Dev 93: 165–168. 10781950 10.1016/s0925-4773(00)00252-5

[41] International HapMap Consortium (2003) The International HapMap Project. Nature 426: 789–796. 14685227 10.1038/nature02168

[42] Horvath S, Xu X, Laird NM (2001) The family based association test method: strategies for studying general genotype–phenotype associations. Eur J Hum Genet 9: 301–306. 11313775 10.1038/sj.ejhg.5200625

[43] Dudbridge F (2008) Likelihood-based association analysis for nuclear families and unrelated subjects with missing genotype data. hum hered 66: 87–98. 18382088 10.1159/000119108 PMC2386559

[44] Rozen S, Skaletsky H (2000) Primer3 on the WWW for general users and for biologist programmers. Methods Mol Biol 132: 365–386. 10547847 10.1385/1-59259-192-2:365

[45] Mansilla MA, Cooper ME, Goldstein T, Castilla EE, Lopez Camelo JS, et al (2006) Contributions of PTCH gene variants to isolated cleft lip and palate. Cleft Palate Craniofac J 43: 21–29. 16405370 10.1597/04-169R.1 PMC2151847

